# Craniofacial changes of ancient populations lived in different eras in Anatolia

**DOI:** 10.1038/s41598-025-06928-4

**Published:** 2025-07-02

**Authors:** Levent Yücel, Ayla Sevim Erol, Salih Cengiz Meral, Fatemeh Azizi, Çilem Sönmez Sözer, Zafer Ünsal Çoşkun, Timur Gültekin, Ceren Karaçaylı, Bülent Satar

**Affiliations:** 1 Department of Otorhinolaryngology, University of Health Sciences, Gülhane Training and Research Hospital, General Dr. Tevfik Sağlam Street, No:1, Etlik, Ankara, 06010 Turkey; 2 Department of Anthropology, Faculty of Humanities, Ankara University, Ankara, Turkey; 3 Department of Radiology, University of Health Sciences, Sultan Abdulhamithan Training and Research Hospital, Istanbul, Turkey

**Keywords:** Craniofacial dimensions, Sex, Era, Ancient population, Anatolia, Evolution, Environmental social sciences

## Abstract

The aim of our study is to determine the changes in craniofacial dimensions among populations lived in Anatolia from different time periods. Eight skulls from the second century AD, 20 skulls were from the tenth–eleventh centuries AD, 20 skulls from the sixteenth–nineteenth centuries AD, and 60 contemporary skulls were included in this cross-sectional observational study. Using computerized tomography scans, five craniofacial dimensions were enrolled: glabella-occipital length (GOL), maximum width (MW), basion-bregma height (BBH), bizygomatic width (BZW), nasion-prosthion height (NPH). The differences of craniofacial dimensions between eras, the effect of sex and/or era on craniofacial dimensions were investigated. The GOL dimensions of the second century AD group were found to be significantly higher than contemporary skulls, and the tenth–eleventh century AD group were also found to be significantly higher than sixteenth–nineteenth century AD group and contemporary skulls (*p* < 0.001). NPH dimensions of tenth–eleventh century AD group were found to be significantly lower than contemporary group (*p* = 0.002). The main effect of sex, era, and sex-era interaction on GOL was found statistically significant (*p* = 0.004, *p* < 0.001, and *p* = 0.017, respectively). Only sex had a statistically significant effect on BZW (*p* < 0.001). Our study showed that GOL has decreased and NPH has increased over the centuries in Anatolian societies. Both sex and era influence the change of GOL.

**Supplementary Information:**

The online version contains supplementary material available at 10.1038/s41598-025-06928-4.

## Introduction

The human skull is one of the most important sources of phylogenetic features and population-based genetic studies, and the complex morphology of the skull is often determined by a series of craniometric measurements. For over 15 years now, complex analyses are also carried out using methods such as statistical shape analysis (geometric morphometrics, among others).

It has been revealed that the human skull has undergone adaptive changes over the centuries in order to adapt to many environmental and genetic modifications, especially environmental adaptation^1^. On the other hand, several studies have shown different factors being almost as important as environmental adaptation, depending on which part of the skull, which is a highly compartmentalized or in other words modular structure^2^. In addition, it has been revealed that sex, economic status, the compared time periods and/or locations are other crucial factors affecting craniofacial changes besides environmental conditions and genetic transmission^3–5^. Due to highly variables, it is not clear that which variable is more crucial on the change of craniofacial morphology.

The main aim of our study was to determine changes of craniofacial dimensions over years among populations lived in Anatolia from different time periods. Our secondary aim was to determine which of gender and age has a greater effect on craniofacial dimensions. As far as we know, this is the first study comparing craniofacial dimensions over years lived in Anatolia.

## Materials and methods

### Demographic descriptors of the ancient samples

The materials belonging to the ancient city of Cyzicus have been dated to the second century AD, and the skeletal remains of four female and eight male individuals found in a sarcophagus in the West Necropolis area to the northwest of the city during excavations in Balıkesir Province, Erdek District, have been dated to the same period. The skeletal remains of one female, three males, and one unidentified individual were recovered from a chamber tomb in the same area that belonged to 17 adult individuals. Among these materials, a total of eight adult skulls (three female and five male) were selected and included in this study.

The materials belonging to the Belentepe rescue excavations have been dated to the 10th–11th centuries AD. They consist of 188 individuals identified from 150 graves unearthed from the Necropolis area of the Eastern Roman period during excavations in the Milas District of Muğla Province. These 188 individuals comprise seven fetuses, 28 infants, 44 children, two adolescents, 33 women, 57 men, and 16 individuals whose sex and/or age could not be determined. The skulls of 20 adult individuals (10 females and 10 males) were selected and included in this study.

The materials obtained from the Karacaahmet Cemetery in Istanbul, which date back to the 16th–19th centuries, consist of the skulls of 20 adult individuals: four females and 16 males. All of them were included in the study.

### Selection of ancient Anatolian skulls

The ancient skulls used in this study were obtained from the Department of Anthropology, Faculty of Language, History and Geography, at Ankara University. Those with a macroscopically preserved appearance were selected and included. Fragmented or damaged skulls were excluded from the study. To compare all measurements between different centuries, skulls from four different centuries were included in the study.

### Selection of contemporary individuals

CT scans of 60 contemporary individuals from the archives of the PACS system were randomly selected, and 29 scans were of women. No pathologies were detected in either the temporal or paranasal sinuses. Skulls with temporal or paranasal pathology were excluded.

In summary, a total of 108 adult skulls from four different eras were included in this study. Distribution of the skulls by sex and era was shown in Table 1.

**Table 1:** Distribution of the skulls by sex and era.

	Female	Male	Total
2nd century AD (Cyzicus)	3	5	8
10th-11th century AD (Belentepe)	10	10	20
16th-19th century AD (Karacaahmet)	4	16	20
Contemporary (Istanbul)	29	31	60
Total	46	62	108

This study was performed in line with the principles of the Declaration of Helsinki. The necessary permissions were obtained from the relevant institution. The institutional ethics committee approved the study (date: June 27, 2022; no: 2022–172) and the study was conducted in line with the related privacy statements and applicable regulatory requirements.

### Imaging using CT

CT scans were obtained using a Canon Aquilion Lightning device (Toshiba Medical Systems, Japan) for the contemporary and ancient skulls, with 1 mm thickness, 200 mA, 120 kV, a 1 s rotation time, and 1 mm interval scanning parameters in the axial, coronal, and sagittal planes. The CT scans of the skulls were numbered, and the axial, coronal, and sagittal images were exported in a DICOM file format.

### Measurements

All measurements were independently performed by two blinded examiners. The examiners (one radiologist at least and/or clinician) did not know the era of any of the CT scans and the data was edited according to eras after finishing all the measurements. CT slices were independently selected by two examiners and slice with maximum dimension was included. In case of disagreement, two examiners calculated separately and decided together on the one with the maximum distance. The interrater reliability of the measurements was assessed using the interrater correlation coefficient (r). Values of r ≥ 0.75 were considered satisfactory (Supplementary Table 1).

All constructions and measurements were performed on a 21.3-inch flat-panel colour active matrix TFT medical display (NEC MultiSync MD215MG, Munchen, Germany) with a resolution of 2048 × 2560 at 75 Hz and 0.17-mm dot pitch operated at 11.9 bits. The examiners were also permitted to use enhancements and orientation tools such as magnification, brightness, and contrast to improve visualization.

**Fig. 1:**
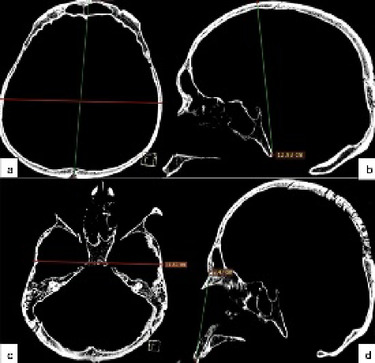
Measurements: (**a**) glabella-occipital length and maximum width. (**b**) basion-bregma height. (**c**) bizygomatic width. (**d**) nasion-prosthion height.

Five craniofacial dimensions were enrolled: glabella-occipital length (GOL) (Fig. 1a), maximum width (MW) (Fig. 1a), basion-bregma height (BBH) (Fig. 1b), bizygomatic width (BZW) (Fig. 1c), nasion-prosthion height (NPH) (Fig. 1d), as similar with Jantz et al.^6^

### Statistical analysis

The data analyzed with IBM SPSS Statistics version 25 (IBM Corp., Armonk, NY, USA). Descriptive statistics were presented as mean ± standard deviation (SD) and median (minimum–maximum) for continuous variables, and frequencies and percentage for categorical variables. Normality hypotheses were tested with Shapiro-Wilks tests. Comparisons of continuous variables between multiple groups were made using One Way ANOVA (using Welch’s correction if necessary) or the Kruskal Wallis test. In the contemporary group, BBH and NPH did not meet the assumptions of normality; therefore, the non-parametric Kruskal–Wallis test was used for their comparisons. Two-way ANOVA was used to examine the effects of era and sex, as well as the interaction between era and sex, on GOL, MB, and BB measurements. Since BBH and NPH did not meet the assumption of normality, the effects of sex and era could not be examined for these measurements. Pairwise comparisons were performed using the Bonferroni correction to control for Type I error across multiple comparisons, without designating a specific control group. A result of *p* < 0.05 was considered statistically significant.

## Results

### Differences in craniofacial dimensions according to eras

A total of 108 adult skulls belong to four different eras were included in the study (Table 1). The descriptive data of the craniofacial dimensions defined before are presented in Table 2.

**Table 2:** Comparison of craniofacial dimensions by eras.

	2nd century AD (n = 8)		10th-11th century AD (n = 20)		16th-19th century AD (n = 20)		Contemporary (n = 60)		Test Stats	*p*
	Mean ± SD	Median (Min–Max)	Mean ± SD	Median (Min–Max)	Mean ± SD	Median (Min–Max)	Mean ± SD	Median (Min–Max)		
GOL	179.29 ± 6.12	179.71 (168.86–188.59)	179.36 ± 10.63	179.3 (161.5–198.9)	172.13 ± 7.12	171.84 (159.89–189)	170.86 ± 8.22	170.4 (152.2–193.5)	6.623	< 0.000*
BBH	139.33 ± 5.01	138.64 (134.09–150.26)	137.44 ± 4.37	136.42 (131.8–147.6)	135.93 ± 6.7	136.01 (122.66–147.28)	139.7 ± 6.82	140.65 (116.7–151.3)	6.878	0.076***
MW	139.67 ± 5.55	140,89 (130.47–146.86)	135.77 ± 6.97	136.1 (115.9–151.9)	139.62 ± 7.38	140.22 (127.08–155.22)	140.68 ± 6.8	140.95 (126.9–158.2)	2.557	0.059*
BZW	129.81 ± 3.67	130.16 (124.14–136.66)	126.94 ± 7.66	125.89 (111.74–140.3)	127.89 ± 7.96	130.04 (108.92–138.97)	128.26 ± 7.07	127.6 (112.9–146.6)	0.341	0.796*
NPH	62.1 ± 3.75	61.81 (57.18–69.19)	61.76 ± 5.05	61.65 (53.5–71.4)	63.06 ± 4.51	63.62 (51.82–69.83)	67.18 ± 13.06	66.45 (38.9–155.8)	14.68	0.002***

*GOL* glabella-occipital length, *BBH* basion-bregma height, *MW* maximum width, *BZW* bizygomatic width, *NPH* nasion prosthion height, *SD* standard deviation. *ANOVA, ***Kruskal Wallis.

Significant values are in bold.

The group comparisons showed no significant difference between 2nd century AD, 10th-11th Century AD and 16th-19th century AD groups in terms of GOL. However, the GOL dimensions of the 2nd century AD group were found to be significantly higher than contemporary skulls, and the values of the 10th-11th century AD group were also found to be significantly higher than 16th-19th century AD group and contemporary skulls (*p* < 0.001, Table 2). No significant difference was observed between the skulls from 16th-19th century AD and contemporary individuals. NPH dimensions of 10th–11th century AD group were found to be significantly lower than contemporary group (*p* = 0.002, Table 2), and no significant difference was detected between the other groups. BBH, BZW, and MW showed no statistically significant difference was found between the groups (*p* > 0.05, Table 2).

### The effect of sex and/or era on craniofacial dimensions

#### Glabella-occipital length

The tested hypotheses for GOL were as follows:

1. There is a main effect of sex on GOL.
2. There is a main effect of era on GOL.
3. There is an interaction between sex and era in determining GOL.

Descriptive statistics for each sex and era group are presented in Table 3. GOL measurements from the 2nd and 10th–11th century AD skulls were generally higher than those from the 16th–19th century AD and contemporary individuals. The highest mean GOL was observed in male skulls from the 10th–11th century AD, while the lowest was found in female skulls from the 16th–19th century AD.

**Table 3:** Descriptive statistics of glabella-occipital length (mm) by sex and era.

	Sex		Total
	Female (n = 46)	Male (n = 62)	
Era			
2nd century AD (n = 8)	182.17 ± 2.58^BC^	177.56 ± 7.23^ABC^	179.29 ± 6.12^a^
10th-11th century AD (n = 20)	171.84 ± 7.45^AB^	186.88 ± 7.59^C^	179.36 ± 10.63^a^
16th-19th century AD (n = 20)	166.9 ± 7.52^A^	173.44 ± 6.61^AB^	172.13 ± 7.12^b^
Contemporary (n = 60)	167.69 ± 7.67^A^	173.82 ± 7.7^AB^	170.86 ± 8.22^b^
Total	169.47 ± 8.15	176.13 ± 8.69	173.29 ± 9.05

F: female, M: male, SD: standard deviation, AD: Anno Domini (Year of the Lord). Two-way ANOVA assumptions of normality and homogeneity of variances were tested and met. Post hoc pairwise comparisons were performed using Bonferroni correction for multiple comparisons. Superscript letters indicate statistically significant differences (*p* < 0.05): a–b: No statistically significant difference between eras containing the same letter. A–C: No statistically significant difference between sex and era combinations containing the same letter. Please refer to Table 4 for the corresponding two-way ANOVA results, including the main and interaction effects of sex and era.

Results from the two-way ANOVA (Table 4) indicated statistically significant main effects of sex (*p* = 0.004) and era (*p* < 0.001), as well as a significant sex × era interaction (*p* = 0.017), confirming that GOL is influenced both by sex and historical period, and that these factors interact in shaping cranial morphology.

**Table 4:** Two-way ANOVA results for the effects of sex, era, and their interaction on glabella-occipital length.

	SS	Df	MS	F	*p*	ηp2
Sex	479.367	1	479.367	8.727	**0.004**	0.080
Era	1565.961	3	521.987	9.503	** < 0.001**	0.222
Sex *Era	586.678	3	195.559	3.560	**0.017**	0.096

Two-way ANOVA. R^2^:0.330, SS: Sum of Squares, df: degree of freedom, MS: Mean of Squares, F: ANOVA F statistic; ηp^2^: partial eta squared (effect size). Significant p-values (*p* < 0.05) are shown in bold.

**Fig. 2:**
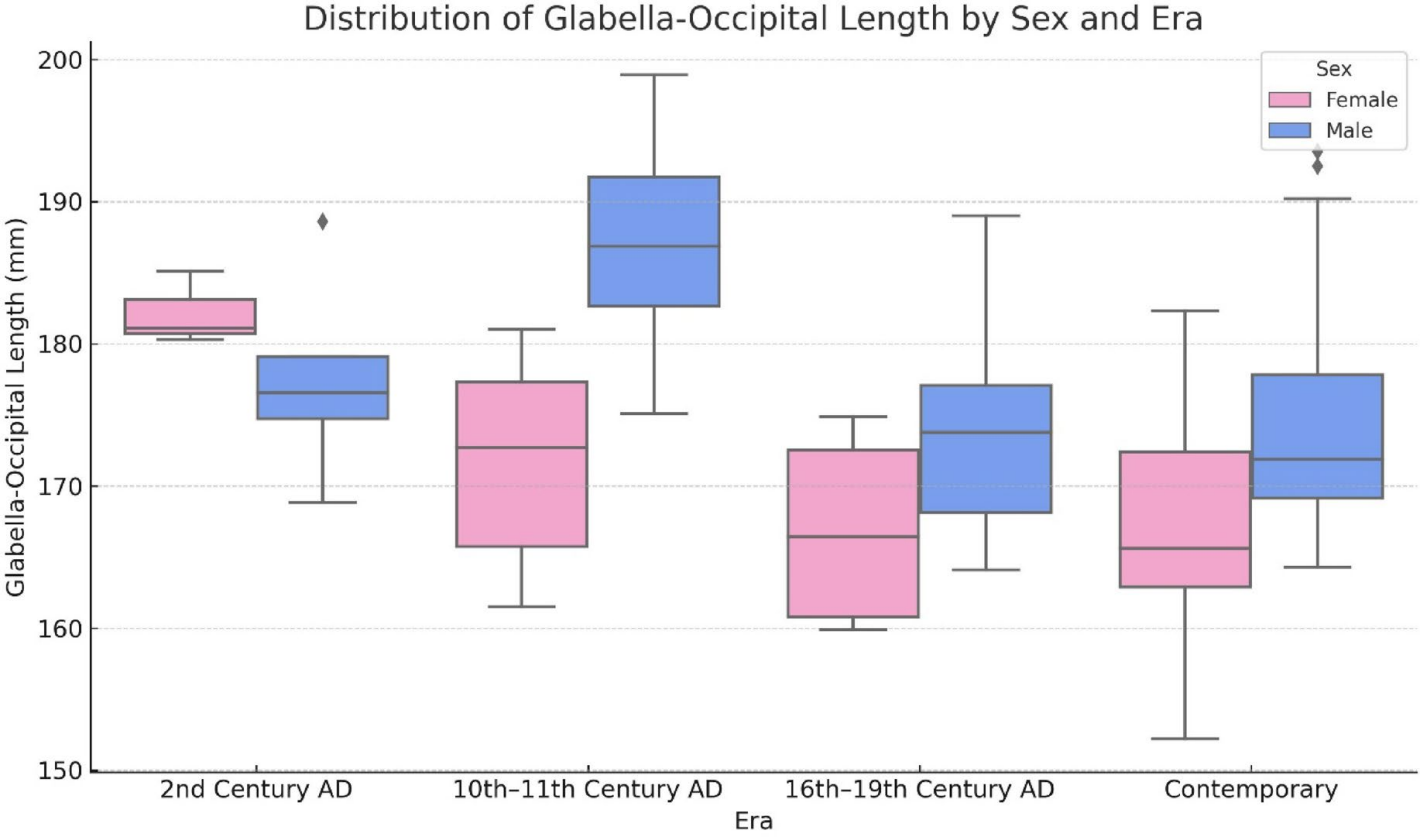
Boxplot showing the distribution of Glabella-Occipital Length (mm) by sex across four historical eras. The figure illustrates differences in central tendency and variability within and between groups.

The distribution of GOL across sex and era groups is visualized in Fig. 2, which complements the statistical findings by showing the group-level differences and variability. The plot clearly illustrates the observed interaction effect (Table 4), particularly the increased cranial length in males from the 10th–11th century AD group.

#### Maximum width

The tested hypotheses for MW were as follows:

1. There is a main effect of sex on MW.
2. There is a main effect of era on MW.
3. There is an interaction between sex and era in determining MW.

Descriptive statistics for each sex and era group are presented in Table 5. The highest average width was observed in male skulls from the contemporary group (142.5 ± 7.54 mm), while the lowest was found in female skulls from the 10th–11th century AD (135.33 ± 3.3 mm).

**Table 5:** Mean ± standard deviation of maximum width (mm) for each sex and era group.

	Sex		Total
	Female	Male	
Era			
2nd century AD (n = 8)	139.02 ± 7.63	140.06 ± 4.92	139.67 ± 5.55
10th-11th century AD (n = 20)	135.33 ± 3.3	136.22 ± 9.56	135.77 ± 6.97
16th-19th century AD (n = 20)	137.36 ± 3.48	140.18 ± 8.05	139.62 ± 7.38
Contemporary (n = 60)	138.73 ± 5.38	142.5 ± 7.54	140.68 ± 6.8
Total	137.89 ± 5.06	140.69 ± 8	139.5 ± 7.01

Two-way ANOVA. Values were given in mean ± standard deviation. Please refer to Table 6 for the corresponding two-way ANOVA results, including the main and interaction effects of sex and era.

However, the two-way ANOVA results (Table 6) showed that neither the main effect of sex (*p* = 0.240) nor era (*p* = 0.060), nor the interaction between sex and era (*p* = 0.845), reached statistical significance.

**Table 6:** Two-way ANOVA results for the effects of sex, era, and their interaction on maximum width.

	SS	df	MS	F	*p*	ηp2
Sex	65,026	1	65.026	1.396	0.240	0.014
Era	356,237	3	118.746	2.550	0.060	0.071
Sex*Era	38,009	3	12.670	0.272	0.845	0.008

Two-way ANOVA. SS: sum of squares, df: degrees of freedom, MS: mean square, F: F-statistic, p: p-value, ηp^2^: partial eta squared (effect size).

**Fig. 3:**
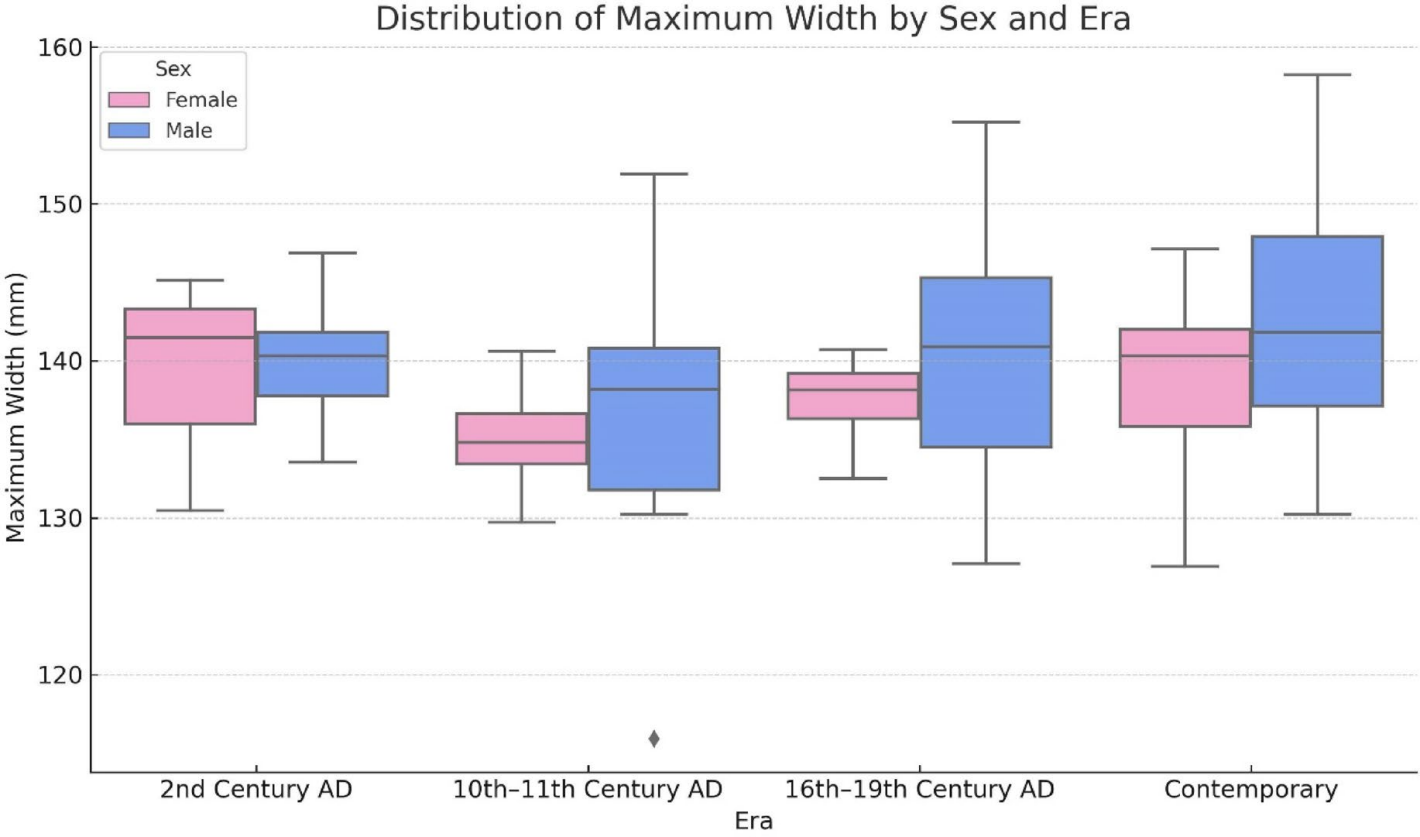
Boxplot showing the distribution of maximum width (mm) across different eras and by sex. Although no statistically significant differences were found in the two-way ANOVA (Table 6), the figure illustrates the variability and central tendency within each group.

The distribution of maximum width values across sex and era groups is illustrated in Fig. 3. Although the two-way ANOVA did not show a statistically significant effect of sex, era, or their interaction (Table 6), the boxplot provides a visual representation of variability and group-level tendencies.

#### Bizygomatic width

The tested hypotheses for BW were as follows:

1. There is a main effect of sex on BW.
2. There is a main effect of era on BW.
3. There is an interaction between sex and era in determining BW.

Descriptive statistics for each sex and era group are presented in Table 7. The highest mean was observed in males from the contemporary group (133.15 ± 5.56 mm), while the lowest was recorded in females from the 16th-19th century AD group (118.39 ± 7.81 mm).

**Table 7:** Mean ± standard deviation of bizygomatic width (mm) for each sex and era group.

	Sex		Total
	Female	Male	
Era			
2nd century AD (n = 8)	127.86 ± 3.78	130.98 ± 3.45	129.81 ± 3.67
10th-11th century AD (n = 20)	123.05 ± 5.46	130.83 ± 7.77	126.94 ± 7.66
16th-19th century AD (n = 20)	118.39 ± 7.81	130.26 ± 6.16	127.89 ± 7.96
Contemporary (n = 60)	123.04 ± 4.17	133.15 ± 5.56	128.26 ± 7.07
Total	122.95 ± 5	131.85 ± 6	128.06 ± 7.11

Two-way ANOVA. SD: standard deviation, AD: Anno Domini (Year of the Lord). Values are presented as mean ± standard deviation.

Two-way ANOVA results (Table 8) revealed a statistically significant main effect of sex (F = 31.823, *p* < 0.001, ηp^2^ = 0.241), indicating that male individuals consistently had greater bizygomatic width than females across all eras. The main effect of era (*p* = 0.118), and the sex × era interaction (*p* = 0.298) were not statistically significant, suggesting that this sex difference remained stable over time. The assumption of homogeneity of variances was met (Levene’s test, *p* > 0.05).

**Table 8:** Two-way ANOVA results for the effects of sex, era, and their interaction on bizygomatic width.

	SS	df	MS	F	*p*	ηp2
Sex	971.966	1	971.966	31.823	< 0.001	0.241
Era	183.988	3	61.329	2.008	0.118	0.057
Sex*Era	113.986	3	37.995	1.244	0.298	0.036

SS: sum of squares, df: degrees of freedom, MS: mean square, F: F-statistic, p: p-value, ηp^2^: partial eta squared (effect size). A statistically significant main effect of sex was observed (p < 0.001), while the effects of era (*p* = 0.118) and the interaction between sex and era (*p* = 0.298) were not statistically significant.

**Fig. 4:**
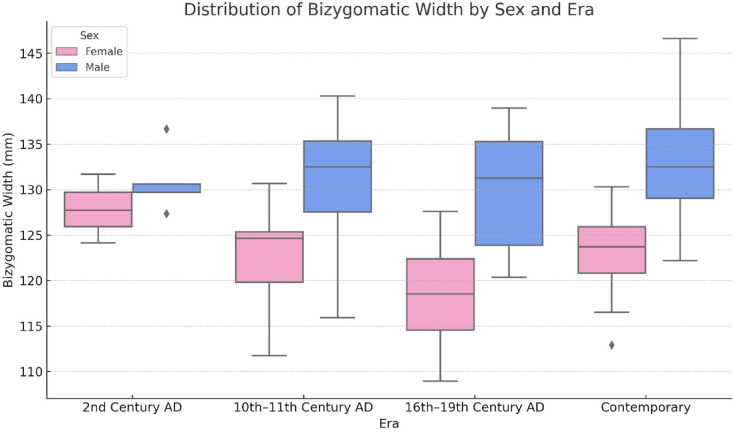
Boxplot showing the distribution of bizygomatic width (mm) by sex across historical eras. The figure illustrates consistently greater values in males compared to females across all periods. Diamonds indicate statistical outliers beyond 1.5 times the interquartile range.

The distribution of BW across sex and era groups is visualized in Fig. 4, which illustrates consistently greater values in males compared to females across all periods. This graphical representation supports the statistically significant main effect of sex while showing no evidence of interaction.

## Discussion

Our study showed that GOL decreased and NPH increased over the centuries in Anatolian societies. In other words, the dolichocephalic appearance has decreased, and the upper face height has increased. Although measurement parameters vary between studies, they compared the craniofacial measurements of the neolithic age and the modern society and found that the GOL decreased, similar to our study, but it was not found to be statistically significant^7^. Similarly, Morant^8^ reported that Neanderthalic skulls had significantly greater GOL compared to modern populations, with La Chapelle specimens measuring over 207 mm, while modern dolichocephalic Europeans and Australians averaged approximately 190 mm and 187 mm, respectively. In their study, Cappabianca et al.^9^ compared modern and ancient Italian population through 2700 and found greater NPH like our study but lower nasion-opisthocranion length, a similar dimension of GOL, in contrast to our study. In another study, NPH decreased in Wu et al.'s study, and they also detected a significant decrease in BW and MW. On the other hand, Argyropoulos^1^ detected that ancient Greece skull had a higher upper face length and a lower anterior cranial fossa length compared to modern skull through 4000 years like our study. As a result, moderate genetic heritable variation may underlie these traits^10^. We believe that this current difference may be many factors such as environmental factors and location, racial characteristics, statistical methods, sample size, compared time period or measurement methods using cephalogram, 2D or 3D CT^1,11^.

Craniofacial evaluations of the skull; it has become a subject of research because it can be applied to the human population living today and in ancient times, shows a high degree of genetic transition, and contains more meaningful changes than other cranial skeletal parts^1^. In addition, recent anthropologic studies have revealed that there have been rapid changes in skull morphology over the past century that humanity has never experienced before^12^. However, it should not be ignored that the changes may arise from method errors, environmental, genetic and other factors. On the other hand, some studies showed that the face and nose shape and the neurocranium are more affected by changing environmental conditions, while the basicranium protects itself more from this effect^3^. Another studies revealed that comparative craniofacial changes in one or two parent lineages are due to environmental causes, while comparative studies covering a longer period are thought to be due to both environmental and genetic changes^13,14^. This may cause studies on craniofacial morphology covering long periods of time, such as our study, to become more remarkable.

Many previous studies based on craniofacial morphological evaluation have contradictory to each other and revealed that there has been an increase or decrease in skull length over the centuries. The hypothesis of studies claiming that there has been an increase in length over time has led to the idea that the increase in brain volume observed over time or conditions related to developmental epigenetic stimulation may be responsible for this increase^9^. Studies showing that skull length has decreased over the centuries have stated that this may be due to the idea that this is an adaptation that occurred in ancient times when the climate was colder^4^. On the other hand, anthropometric studies comparing similar periods show that the skull’s tendency to brachycephalization (e.g. Japanese, Koreans and Mexicans) has increased in some societies until today, and to dolichocephalization (e.g. Americans and Croatians) in some societies^6,15–18^.

In a study using data from the Korea Research Institute of Standards and Science and many ancient skulls from different times, it was observed that the tendency to brachycephalization has increased in the Korean population for approximately 2000 years until today, like our study, and this has become evident in the last 100 years. However, the fact that a transition to debrachycephalization has been observed in the last 50 years, as in the British and Japanese populations, supports that cranial morphology has changed more rapidly in recent times, as stated before^8,12,17,19–21^. Although many theories have been put forward to explain this debrachycephalization, the most accepted theory is the improvement in nutrition and health status^6,15^. In another theory, the craniofacial changes observed in men are more pronounced and that men may have developed a greater developmental plasticity against environmental changes than women. Although the reason for this situation has not yet been fully confirmed, men may respond faster to economic changes than women^12,22^. In summary, the literature has no consensus on how the craniofacial changes exist due to many affecting variables.

This study has some limitations. Firstly, the study has small numbers of skulls. Secondly, although all the studied populations had lived in Anatolia, the fact that the skulls were from different regions of Anatolia is another limitation of our study. Thirdly, as we did not use 3D reconstruction method to calculate the dimensions, some minor errors must be taken into account when evaluating our results. However, we believe that comparing four populations that lived approximately 500, 1000, and 2000 years apart is the strength of our study. Future investigations with larger populations that lived in the same or nearby regions with similar genetic characteristics will more clearly reveal the effect of time on the parameters evaluated in our study.

## Conclusion

Our study showed that GOL has decreased and NPH has increased over the centuries in Anatolian societies. Both sexes and era influence the change of GOL.

## Electronic supplementary material

Below is the link to the electronic supplementary material.

Supplementary Material 1

## Data Availability

Data are available upon request from the corresponding author.
