# Supplementary material for: Craniofacial changes of ancient populations lived in different eras in Anatolia
Source: Sci Rep. 2025 Jul 2;15:23458. doi: 10.1038/s41598-025-06928-4 (PMC12223272; doi:10.1038/s41598-025-06928-4)
Supplement: Supplementary file 1 — Supplementary Material 1 [file 41598_2025_6928_MOESM1_ESM.docx]

**Supplementary Table 1.** Correlation matrix analysis of the variables.

|  | **Glabella-occipital length** | **Basion-bregma height** | **Maximum breadth** | **Bizygomatic breadth** | **Nasion-prosthion height** |
| --- | --- | --- | --- | --- | --- |
| **Glabella-occipital length** | 1 |  |  |  |  |
| **Basion-bregma height** | 0,185 (ρ) | 1 |  |  |  |
| **Maximum breadth** | 0,041(r) | 0,147(ρ) | 1 |  |  |
| **Bizygomatic breadth** | 0,318 (r) | 0,394 (ρ) | 0,451 (r) | 1 |  |
| **Nasion-prosthion height** | 0,080 (ρ) | 0,404 (ρ) | 0,110 (ρ) | 0,266 (ρ) | 1 |

r: Pearson correlation coefficient, ρ: Spearman correlation coefficient
